# 

**Published:** 1905-09-16

**Authors:** 


### NOTICE TO CORRESPONDENTS.

All MSS., Letters, Books for Review, and other matters intended for the Editor should be addressed THE EDITOR, "THE HOSPITAL," THE HOSPITAL BUILDINGS, 28 & 29 SOUTHAMPTON STREET, STRAND, W.C.

The Editor cannot undertake to return rejected MS., even when accompanied by stamped directed envelope.

**Notice to Advertisers and Subscribers.**

All Advertisements, Orders for Copies of THE HOSPITAL, and Business Communications should be addressed to **THE MANAGER (Not to the Editor),** THE HOSPITAL, 28 & 29 Southampton Street, Strand, London, W.C.

The Cost of Subscription, post free, is at follows:—Per week, 3½d.; per quarter, 3s. 3d.; per half-year, 6s. 6d.; per annum, 13s. Foreign and Colonial:—Per annum, 19s. 6d., payable, in all cases, in advance.

**NOTICE.—Vol. XXXVII. of "The Hospital" October 1904 to March 1905), in handsome cloth binding, will shortly be on sale at the office of this Journal, price 10s. 6d. Cases for binding the Numbers contained in this Volume 2s. Index to Vol. XXXVII., 3½d., post free.**

**The Monthly Part for September is now ready. Price 1s., by post, 1s. 3d.**

### Medical Appointments.

**Leonard Crossley**, M.D.Edin., Senior Resident Medical Officer to the Royal National Hospital for Consumption, Ventnor, Isle of Wight. **John Drew**, M.D.Glasg., Certifying Surgeon under the Factory and Workshop Act for the Stirling District of the county of Stirling. **Francis H. Ellis**, B.A., M.B., B.C.Cantab., M.R.C.S.Eng., L.R.C.P.Lond., Assistant Resident Medical Officer at the London Open-air Sanatorium, Pinewood, Wokingham, Berks. **T. Parker Greenwood**, M.D., B.Sc., Assistant Medical Officer at the County Asylum, Radcliffe, Notts. **Frederick W. Kemp**, M.B., B.Ch.Durh., M.R.C.S.Eng., L.R.C.P.Lond., Junior House Surgeon at the Radcliffe Infirmary, Oxford. **Thomas Knowles**, M.D.Edin., Certifying Surgeon under the Factory and Workshop Act for the Gargrave District of the county of York. **H. C. Lecky**, M.B., B.Ch.Oxon., House Physician at the Radcliffe Infirmary, Oxford. **John W. Mackenzie**, M.D.Edin., Assistant Member of the Medical Staff of the Northern Infirmary, Inverness. **Peter McDermid**, M.B., Ch.B., has been appointed Junior Physician to the Lancashire County Asylum, Winwick. **Sheffield Neave**, M.R.C.P.Lond., M.R.C.S.Eng., Assistant Physician to the North-Eastern Hospital for Children. **William L. Norwood**, L.R.C.P., L.R.C.S.Edin., L.F.P.S.Glasg., Certifying Surgeon under the Factory and Workshop Act for the Bellanagh District of the county of Cavan. **Frederick W. Price**, M.B., M.R.C.P., Medical Registrar to Westminster Hospital **Thomas H. S. Pullin**, M.D.St.And., F.R.C.S.Edin., M.R.C.S. Eng., L.S.A., re-appointed Medical Officer of Health of Sidmouth, Devon, for three years. **Augustus Keppel Reede**, L.R.C.P., L.R.C.S.Edin., L.A.H.Dub., Medical Officer and Public Vaccinator for the Upottery and Dunkeswell Districts by the Honiton (Devon) Board of Guardians. **James R. Marr Richmond**, L.S.A., Medical Officer of Health for the West Penwith (Cornwall) Rural District. **Edwin W. Routley**, M.D.Brux., D.P.H.Cantab., M.R.C.S.Eng., L.R.C.P.Lond., Medical Officer of Health of Aldershot and Medical Superintendent of the Isolation Hospital, North Town, Aldershot.

### THE SIGN of a Good Nursing Book.

### A SELECTION

**THE NURSING PROFESSION: How and Where to Train.**
By Sir HENRY BURDETT, K.C.B. **2/-** net, **2/4** post free.
A Complete Guide to Training for the Nursing Profession.

**NURSING: ITS THEORY AND PRACTICE.**
By Dr. PERCY LEWIS. **3/6** post free.
A Complete Text-book of Medical, Surgical, and Monthly Nursing.

**THE NURSES' PRONOUNCING DICTIONARY of Medical Terms and Nursing Treatment.**
By HONNOR MORTEN. Revised Edition by Mary I. Burdett. **2/-** post free.
An indispensable Reference Book for Nurses. Suitable size for the apron pocket.

**MODERN NURSING OF CONSUMPTION.**
By Dr. JANE WALKER. **1/-** net, **1/2** post free.
A Valuable Guide to Nursing of "Open-Air" Cases.

**SURGICAL WARD-WORK AND NURSING.**
By Dr. ALEX. MILES. **3/6** net, **3/10** post free.

**A COMPLETE HANDBOOK OF MIDWIFERY.**
By J. K. WATSON, M.D. **6/-** net, **6/4** post free
Profusely Illustrated.

**PRACTICAL GUIDE TO SURGICAL BANDAGING AND DRESSINGS.**
By WM. JOHNSON SMITH, F.R.C.S. **2/-** post free.
This work has been especially designed for Nurses by a surgeon of great experience.

**SIMPLE SANITATION: The Practical Application of the Laws of Health to Small Dwellings.**
By Miss M. LOANE. **1/-** net; **1/2** post free.

**AN ELEMENTARY TREATISE ON THE LIGHT TREATMENT.**
By Dr. JAMES H. SEQUEIRA. **2/6** net, **2/9** post free.

**HINTS TO NURSES ON TROPICAL FEVERS.**
By Dr. F. POLLARD. **1/6** net, **1/8** post free.
A work of the utmost value to Nurses and to all residents in the Tropics.

**The Scientific Press, Ltd.**

*Complete Catalogue sent post free on application.*

**Publishers of Nursing Text-Books, Charts, and Case-Books of all descriptions.**

**28 & 29 SOUTHAMPTON STREET, STRAND, LONDON, W.C.**

